# Effects of respiratory training on ventilation potential and extubation time in patients with tracheotomy: A protocol for systematic review and meta-analysis

**DOI:** 10.1097/MD.0000000000027704

**Published:** 2021-11-19

**Authors:** Tingting Hu, Jianmei Jiang, Xiaoling Deng, Wei Xiang, Chuan Tan

**Affiliations:** The Central Hospital of Enshi Tujia and Miao Autonomous Prefecture, Enshi, Hubei, China.

**Keywords:** meta-analysis, protocol, respiratory training, tracheotomy

## Abstract

**Background:**

In the early treatment of critical patients, tracheotomy is often performed to improve ventilation. Clinical practices usually start respiratory training after the artificial airway is removed. It is still controversial whether respiratory training during tube occlusion has effects on patients’ ventilation potential and tube occlusion time. Therefore, this study used Meta-analysis to evaluate the effects of respiratory training on patients’ ventilation potential and tube occlusion time during tracheotomy in patients with tube occlusion, so as to provide evidence for rehabilitation treatment.

**Methods:**

Wanfang, Chinese Biomedical Literature Database, Chinese National Knowledge Infrastructure, the Chongqing VIP Chinese Science and Technology Periodical Database, PubMed, Embase, and Web of Science were searched through the computer and the randomized controlled trials of respiratory training on ventilation potential and extubation time in patients experiencing tracheotomy were collected as well. The searching time is limited to October 2021. The language restrictions are Chinese and English. Meta-analysis is performed by using RevMan5.4.

**Results:**

The results of this meta-analysis will be submitted to a peer-reviewed journal for publication.

**Conclusion:**

This study will provide the latest evidence for the rehabilitation training of patients who underwent tracheotomy.

**OSF Registration Number:**

DOI 10.17605/OSF.IO/6UCQF.

## 1 Introduction

Tracheotomy is an important method to rescue acute and critical patients, and it can relieve respiratory obstruction, keep the respiratory tract unobstructed, and maintain the stability of vital signs.^[1–3]^ Due to various factors, including diseases and disuse atrophy of diaphragm that is associated with brake or ventilator, after extubation, some patients still have complications such as lung infection, dyspnea. Meanwhile, after extubation failure, another tracheotomy or intubation increases the pain and economic burden of patients.^[4]^ Therefore, it is particularly important to carry out rehabilitation training for patients with tracheotomy.

Respiratory training can improve the function of the diaphragm and respiratory muscle outside the diaphragm, improve the endurance of respiratory muscle, and is widely used for patients with respiratory dysfunction that is caused by various factors.^[5–7]^ Results of meta-analysis made by Yun et al^[8]^ showed that respiratory training can improve lung function in patients with chronic obstructive pulmonary disease. Pozuelo-Carrascosa et al^[9]^ proved that breathing training can effectively improve respiratory function and walking ability in patients after stroke. Wang et al^[10]^ revealed that respiratory training can reduce lung complications after lung cancer surgery and shorten the length of hospital stay in patients with lung cancer. Therefore, respiratory training is of great significance in the rehabilitation treatment of pulmonary diseases.

Respiratory training usually involves patient breathing through nose and mouth. Therefore, respiratory training is routinely performed only after the artificial airway is removed. However, it is still controversial whether respiratory training during tube occlusion has any effects on patients’ ventilation potential and tube occlusion time.^[11]^ Therefore, this study carried out meta-analysis to evaluate the effects of respiratory training on patients’ ventilation potential and tube occlusion time during tracheotomy in patients with tube occlusion, so as to provide evidence for rehabilitation treatment.

## 2 Methods

### 2.1 Study registration

The protocol of the systematic review has been registered on Open Science Framework, with a registration number of DOI 10.17605/OSF.IO/6UCQF. This meta-analysis protocol is based on the preferred reporting items for systematic reviews and meta-analysis protocols statement guidelines.^[12]^

### 2.2 Data sources and search strategies

Wanfang, Chinese Biomedical Literature Database, Chinese National Knowledge Infrastructure, the Chongqing VIP Chinese Science and Technology Periodical Database, PubMed, Embase, and Web of Science were searched and the randomized controlled trials (RCTs) of respiratory training on ventilation potential and extubation time in patients with tracheotomy were collected as well; The retrieval time is from the establishment to October 2021. The language restrictions were Chinese and English. The search strategy for PubMed is in Table 1.

**Table 1:** Search strategy of the PubMed.

Number	Search terms
#1	Tracheotomy[MeSH]
#2	Tracheotomies[Title/Abstract]
#3	or/1–2
#4	Respiratory rehabilitation training[Title/Abstract]
#5	Breathing exercise[Title/Abstract]
#6	Breath training[Title/Abstract]
#7	Respiratory training[Title/Abstract]
#8	or/4–7
#9	Randomized Controlled Trial[MeSH]
#10	Random∗[Title/Abstract]
#11	Clinic trial [Title/Abstract]
#12	or/9–11
#13	#3 and #8 and #12

### 2.3 Inclusion criteria for selection of studies

The inclusion criteria for this study are as follows: Participants: Tracheotomy patients meeting the standard of Plugging occlusion; Type of study: RCTs; Intervention measures: The control group underwent routine pipe plugging process, and the experimental group received respiratory training on the basis of the control group; Outcome measures: Forced vital capacity, deep inspiratory capacity and plugging Time.

The literature exclusion criteria are as follows:

Review, retrospective studies, and repeatedly published literature.

### 2.4 Data collection and analysis

**Figure 1:**
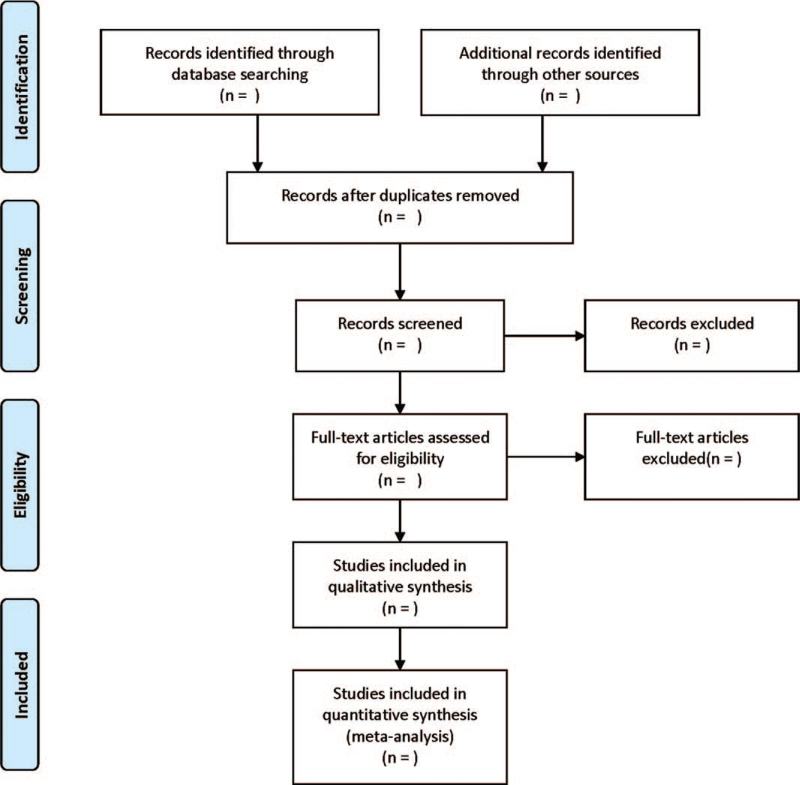
PRISMA flow diagram of the study selection process.

The literature screening process is displayed in Figure 1. Two reviewers independently screened the titles and abstracts of papers and selected the relevant studies. Two same reviewers independently extracted the data from the studies according to a pre-specified protocol, with any disagreement being settled by a third reviewer. The following items were extracted: name of the first author, publication year, type of disease, sample size, duration of respiratory training, outcome measures, and so on.

### 2.5 Assessment of the quality of the included studies

The risk of bias assessment was performed using the Cochrane Collaboration tool. The assessment includes random allocation method, allocation concealment, blind method, integrity of outcome data, selective reporting of research results, and other biases.

### 2.6 Management of missing data

In case any data were missing, we e-mailed the corresponding author of the original article and asked if they could provide the required information.

### 2.7 Statistical analysis

RevMan5.4 software (Cochrane Collaboration, London, United Kingdom) will be used for analysis. Continuous variable data were represented by standardized mean difference and 95% confidence intervals. Cochran *Q* test and the *I*^2^ statistical test were conducted to assess the statistical heterogeneity of the pooled results. If *P* > .10 and *I*^2^ ≤ 50%, there is no statistical heterogeneity among the studies, and the fixed effects model is adopted for meta-analysis; if *P* ≤.10 and *I*^2^ > 50%, there is a high degree of heterogeneity among the studies, and the random effects model is applied for meta-analysis. When the heterogeneity is too large or unable to find the data source, the meta-analysis will be abandoned and only descriptive analysis is made.

### 2.8 Additional analyses

#### 2.8.1 Subgroup analysis

A subgroup analysis will be made on the basis of the duration of respiratory training, type of disease, and sample size.

#### 2.8.2 Sensitivity analysis

The sensitivity of each index will be analyzed by adopting elimination method to test the stability of the results.

#### 2.8.3 Reporting bias

A funnel plot was used to assess potential publication bias.^[13,14]^

### 2.9 Ethical review and informed consent of patients

The contents of this article do not involve moral approval or ethical review and will be presented in print or at relevant conferences.

## 3 Discussion

Respiratory training increases the strength of inspiratory muscle and breathing muscle, vital capacity and oxygen absorption capacity through over-force inspiratory training, and exhalation training.^[15–17]^ The 3 trainings are beneficial to improve the lung ventilation function of patients and reduce the incidence of pulmonary complications. In this study, RCTs of ventilation potential and tube occlusion time of patients undergoing respiratory training during tracheotomy were meta-analyzed, so as to provide evidence-based medicine evidence for clinical work and better guide clinical work.

## Author contributions

**Data curation:** Jianmei Jiang.

**Formal analysis:** Jianmei Jiang.

**Methodology:** Xiaoling Deng.

**Project administration:** Tingting Hu.

**Supervision:** Tingting Hu.

**Validation:** Wei Xiang.

**Visualization and software:** Wei Xiang.

**Writing – original draft:** Chuan Tan and Tingting Hu.

**Writing – review & editing:** Chuan Tan and Tingting Hu.
